# The Putative Thiosulfate Sulfurtransferases PspE and GlpE Contribute to Virulence of *Salmonella* Typhimurium in the Mouse Model of Systemic Disease

**DOI:** 10.1371/journal.pone.0070829

**Published:** 2013-08-05

**Authors:** Inke Wallrodt, Lotte Jelsbak, Lotte Thorndahl, Line E. Thomsen, Sebastien Lemire, John E. Olsen

**Affiliations:** 1 Department of Veterinary Disease Biology, Faculty of Health and Medical Sciences, University of Copenhagen, Copenhagen, Denmark; 2 Department of Systems Biology, Technical University of Denmark, Lyngby, Denmark; University of Birmingham, United Kingdom

## Abstract

The phage-shock protein PspE and GlpE of the glycerol 3-phosphate regulon of *Salmonella enterica* serovar Typhimurium are predicted to belong to the class of thiosulfate sulfurtransferases, enzymes that traffic sulfur between molecules. In the present study we demonstrated that the two genes contribute to *S*. Typhimurium virulence, as a *glpE* and *pspE* double deletion strain showed significantly decreased virulence in a mouse model of systemic infection. However, challenge of cultured epithelial cells and macrophages did not reveal any virulence-associated phenotypes. We hypothesized that their contribution to virulence could be in sulfur metabolism or by contributing to resistance to nitric oxide, oxidative stress, or cyanide detoxification. *In vitro* studies demonstrated that *glpE* but not *pspE* was important for resistance to H_2_O_2_. Since the double mutant, which was the one affected in virulence, was not affected in this assay, we concluded that resistance to oxidative stress and the virulence phenotype was most likely not linked. The two genes did not contribute to nitric oxid stress, to synthesis of essential sulfur containing amino acids, nor to detoxification of cyanide. Currently, the precise mechanism by which they contribute to virulence remains elusive.

## Introduction

The Gram-negative bacterium *Salmonella enterica* serovar Typhimurium (*S*. Typhimurium) is a major pathogen of both animals and humans. It invades epithelial cells of the small intestine and causes inflammation of this organ, usually leading to a self-limiting gastroenteritis [1]–[2]. In mice, the bacterium causes a typhoid-like systemic disease. Important features of this manifestation include the ability to invade in the intestine, to infect and kill macrophages, to survive and replicate within dendritic cells and macrophages and to spread to the reticulo-endothelial system of organs such as liver and spleen [1]–[2]. In order to do so, *S*. Typhimurium possesses several virulence factors that are often encoded as gene clusters on so called *Salmonella* pathogenicity islands (SPIs). Two of the major SPIs, SPI-1 and SPI-2, encode type three secretion systems (T3SSs) that inject effector molecules into the host cell to mediate the invasion process and intracellular survival [3].

*Salmonella* has to cope with several stress conditions during the infection process [4]–[5]. Nitric oxide (NO) stress caused by the release of NO and reactive nitrogen species (RNS) is one such stress factor [6]–[10]. NO and RNS nitrosylate and inactivate reactive metal centers and iron-sulfur clusters, thereby inhibiting the functionality of key bacterial enzymes, such as metabolic, respiratory and DNA synthesizing proteins [6]–[10]. Thus to carry out the infection, *Salmonella* has to activate several defense mechanisms to detoxify NO and RNS, and to repair the damages that they cause [6], [10]–[11]. Oxidative stress is another host defense mechanism that *Salmonella* has to overcome during infection, and several stress systems specifically deal with detoxification of oxygen radicals [4].

Sulfurtransferases shuffle sulfur between molecules [12]–[13]. The enzymes have a carboxy-terminal domain carrying an active-site cysteine, which is important for the sulfur transport [12]. Among other functions, sulfurtransferases are believed to be involved in sulfur metabolism [14]–[15], cyanide detoxification [16]–[17] and the repair and assembly of the iron-sulfur clusters mentioned above as targets for the damaging effects of NO and RNS [13], [18]–[21].

PspE and GlpE are single-domain thiosulfate sulfurtransferases (TSTs), which have been demonstrated *in vitro* to have rhodanese activity in *Escherichia coli* [22]–[24]. The enzymes can detoxify cyanide; however, other substrates such as dithiols may also be utilized by these enzymes [23]–[24]. Recently PspE has been categorized in *E. coli* as a periplasmic rhodanese. It was shown to contribute to the restoration of disulphide bond formation in proteins in the cell envelope in a DsbA mutant in conjunction with the protein DsbC [25]. No function has yet been attributed to these two proteins in *Salmonella*.

PspE is a member of the phage-shock protein (Psp) system that responds to membrane stress (reviewed in [26]–[27]). Expression of *pspE* in *S.* Typhimurium occurs together with the other genes of the *pspABCDE* operon from the σ^54^-dependent *psp* promoter [28]. However, *pspE* expression is likely to happen from an intrinsic, *pspE*-specific σ^70^-dependent promoter [29], as shown for *E. coli* [30]. Expression of *pspE* is highly induced during infection of eukaryotic cells [31]–[32], which indicates a role in host pathogen interaction.

*glpE* is a member of the *glpEGR* operon [33]. Transcription in *E. coli* has been shown to occur from a cyclic AMP-cAMP receptor protein (cAMP-CRP) complex-dependent promoter, generating one polycistronic *glpEGR* mRNA [34]. Furthermore, *glpG* and/or *glpR* genes are transcribed from three additional promoters [33]. GlpR is a repressor of the glycerol 3-phosphate regulon, thus involved in the metabolism of glycerol 3-phosphate and its precursors [35]. However, *glpE* might function independent of the other members of the *glp* regulon, as it does not contribute to the metabolism of glycerol 3-phosphate in *E. coli* [22], [24]. In *E. coli* the cytoplasmic protein GlpE and the periplasmic protein PspE show functional redundancy and together they are responsible for 95% of the thiosulfate sulfurtransferase activity [24].

Given that *pspE* is highly expressed in *S.* Typhimurium during infection [31]–[32], and given that GlpE and PspE seem to have overlapping functions in *E. coli* [24], we hypothesized that their combined activity might be important for virulence of *S.* Typhimurium. We demonstrated that this is indeed the case, and that virulence in a mouse model was affected when both genes were inactivated, but not when single genes were knocked-out. Despite further studies using cell culture models and different *in vitro* growth and survival assays, however, we failed to identify the mechanism by which these proteins contribute to virulence.

## Materials and Methods

### Bacterial Strains and Growth Conditions

Bacteria used in this study are listed in Table 1. Deletion of single genes with parallel insertion of a resistance cassette in *S*. Typhimurium 4/74 was performed using the Lambda Red recombination system as described [38]. Sequences of oligonucleotides used for Lambda Red mediated mutagenesis and PCR verifications are listed in Table 2. Insertions were confirmed by PCR and sequencing, using standard procedures. Phage P22HT105/int^−^201-mediated transduction was performed as described previously [41] to transfer mutations to a clean 4/74 background and to generate double knockout mutants.

**Table 1:** Bacterial strains and plasmids used in this study.

Strain or plasmid	Genotype	Reference or source
**JEO3774**	*S.* Typhimurium 4/74 wild type	[36]
**JEO4560**	*S.* Typhimurium 4/74 ΔglpE::Kan (kan^r^)	This work
**JEO4865**	*S.* Typhimurium 4/74 ΔglpE::Kan+pINS05 (kan^r^ amp^r^)	This work
**JEO4835**	*S.* Typhimurium. 4/74 ΔpspE::Cm (cm^r^)	This work
**JEO4867**	*S.* Typhimurium 4/74 ΔpspE::Cm+pINS02 (cm^r^, amp^r^)	This work
**JEO4836**	*S.* Typhimurium 4/74 ΔglpE::Kan/ΔpspE::Cm (kan^r^, cm^r^)	This work
**JEO4869**	*S.* Typhimurium 4/74 ΔglpE::Kan/ΔpspE::Cm +pINS04 (kan^r^, cm^r^, amp^r^)	This work
**JEO3775**	*S.* Typhimurium 4/74 ΔinvH201::Tn*phoA*	[37]
**pKD3**	Red template for amplification of Cm resistance cassette (amp^r^, cm^r^)	[38]
**pKD4**	Red template for amplification of Kan resistance cassette (amp^r^, Kan^r^)	[38]
**pKD46**	Vector for Lambda Red mediated mutagenesis; λ-Red expression from arabinose-inducible promoter; temperature sensitive (amp^r^)	[38]
**pACYC177**	Cloning vector (amp^r^, kan^r^)	[39]
**pINS02**	pspE in pACYC177 (amp^r^)	This work
**pINS04**	glpE and pspE in pACYC177(amp^r^)	This work
**pINS05**	glpE in pACYC177 (amp^r^, kan^r^)	This work
***E. coli*** ** TOP10**	Chemically competent *E. coli* cloning strain	Invitrogen
**KP1274**	*S*. Typhimurium LT2 (restriction-deficient)	[40]

**Table 2:** Oligonucleotide sequences for PCR based amplification and sequencing.

Primer	Sequence	Application
**glpE**	*for*: 5′GCCTTAATTGGCTTCACCGGCAATAATGAAAGAGCGATTCTGTGTAGGCTGGAGCTGCTTC′3	Lambda Red recombination
	*rev*: 5′ CCCTTCTCGTCGGAGGCTGCATCTTTATGGGATTACGCGCATATGAATATCCTCCTTAG′3	
**pspE**	*for*: 5′TGCGTTAGCGTTATTCGTAGCCATGCCGCTTTATGCCGCATGTGTAGGCTGGAGCTGCTTC′3	Lambda Red recombination
	*rev*: 5′CGGCATATCAAGACGACTGATGCCGCCCATATTCATCGCGCATATGAATATCCTCCTTAG′3	
**glpE_C**	*for*: 5′ACGCGCTATTGGTAACATCG′3	Proof of Lambda Red mutation
	*rev*: 5′CCTGGGTGGCCATGTAATCA′3	
**pspE_C**	*for*: 5′CTGGAGCCAGTACTTCGTAAG′3	Proof of Lambda Red mutation
	*rev*: 5′GCAGCCTGCGTTATAGATATGTGC′3	
**glpE_BamHI**	*for*: 5′ATGGATCCCAGACGGAACTCGTAGTGCT′3	Complementation
	*rev*: 5′ATGGATCCGAGGATTCGCAAAGGAAGTA′3	
**pspE_HindIII**	*for*: 5′ACTGAAGCTTGCTACGTCACGTCAGATACG′3	Complementation
	*rev*: 5′ACTGAAGCTTACTACCCGGAGAGATCAACA′3	
**pACYC177_BamHI**	*for*: 5′CGGTTCGGTTTATTGACGAC′3	Proof of insertion
	*rev*: 5′ACCAGTGCCCTTCTGATGAA′3	
**pACYC177_HindIII**	*for*: 5′CGTATTTCGTCTCGCTCAG′3	Proof of insertion
	*rev*: 5′CGACTCGTCCAACATCAATA′3	
**hilA _q**	*for*: 5′AACACTGTACGGACAGGGCTATCGG′3	qPCR [42]
	*rev*: 5′TACCATCGGGTATCATCTGCCCGGA′3	
**invG_q**	*for*: 5′GGAACAATGATCCGAGTGCT′3	qPCR
	*rev*: 5′ACGCATCTACGGAGGTGGTA′3	
**prgH_q**	*for*: 5′GTCCTGTGCGGTAATCTGCT′3	qPCR
	*rev*: 5′ATGGAAACTCACAGCCGTTC′3	
**sopB_q**	*for*: 5′ACTCAGCAGCAGGATGGCTTACCTG′3	qPCR [42]
	*rev*: 5′TCATGCACACTCACCGTGGACATCC′3	
**pspE_q**	*for*: 5′GCCGCAGAATACTGGATAGATG′3	qPCR
	*rev*: 5′TCCTGTCTGGAACTACCGTTTC′3	
**glpE_q**	*for*: 5′AGAGGCGTATCAGAAACTGCAC′3	qPCR
	*rev*: 5′CCAGCGTATCGTTAGTCAAGTG′3	
**rsmC_q2**	*for*: 5′GAAAAGCAGCCGCAGTTTAG′3	qPCR
	*rev*: 5′CAGTTGGCTACCAACATCCA′3	
**nusG_q2**	*for*: 5′GTCCGTTCGCAGACTTTAAC′3	qPCR
	*rev*: 5′GCTTTCTCAACCTGACTGAAG′3	

Strains were maintained in LB-Lennox broth (LB). For growth on solid media, LB was enriched with 1.5% agar producing LB agar plates. If not stated otherwise, bacteria were grown in M9 minimal salt medium (containing per liter: 12.8 g Na_2_HPO_4_-12H_2_0, 3.0 g KH_2_PO_4_, 0.5 g NaCl, 1.0 g NH_4_Cl, 0.1 mM CaCl_2_, 2 mM MgSO_4_ and 0.4% Glucose) at 37°C, 200 rpm for 16–18 h. When necessary, media was supplemented with antibiotics at the following concentration: 100 µg ampicillin ml^−1^, 50 µg kanamycin ml^−1^ and 10 µg chloramphenicol ml^−1^.

### Construction of Complementation Plasmids

*glpE* and *pspE-*specific PCR products plus their upstream located promoter regions (approx. 400 bp) were cloned into pACYC177 [39] following standard procedures. Oligonucleotides used for construction of complementation plasmids and verificatoin of insertions are listed in Table 2. The constructs were transformed into One Shot® *E. coli* TOP10 chemically competent cells following the recommendations given by the supplier (Invitrogen). Insertion of *glpE* and *pspE* was confirmed by PCR and sequencing. The plasmids were further transformed into KP1274 [40], a restriction-deficient *Salmonella* strain, and finally to *glpE* and *pspE* mutant strains to test for genetic complementation. Expression of *glpE* and *pspE* genes from the complementation plasmids was confirmed by qPCR (see method below).

### RNA Extraction and qPCR

Bacteria were grown to logarithmic phase in M9 (OD_600nm_ = 0.4±0.01). RNA was isolated from 1.5 ml aliquots by mechanical disruption with the FastPrep system (Bio101; Q-biogene) and help of the RNeasy mini kit (Qiagen). Quantity and quality of total RNA was checked with the NanoDrop 1000 spectrophotometer (Thermo Scientific) and on a 1.5% (w/v) agarose gel. All enzymatic steps described below were performed according to the supplier’s recommendation (Fermentas). The RNA was DNase treated with the RNase free DNaseI kit and reverse transcribed with the RevertAid H minus reverse transcriptase kit. The qPCR was done with the Maxima SYBR Green/Rox qPCR Master Mix and gene specific oligonucleotides (Table 2) in a MxPro3000 cycler (Stratagene). qPCR was performed in parallel in samples of the wild type and mutant strains. Data were normalized against two reference genes, *rsmC* and *nusG* [32], [43], producing similar results. Relative gene expression (change fold = CF) in mutant strains compared to the wild type, was calculated by help of the 2^−ΔΔCT^ method [44] corrected by the different primer efficiencies [45].

### The Contribution of the Gene Products to Sulfur Metabolism

The ability to grow in M9 media which contains MgSO_4_ as the only sulfur source was measured. Bacteria from exponentially growing cultures in LB were collected by centrifugation, washed in PBS and re-suspended in the M9 medium at an OD_600_ value of 0.005. The contribution of the enzymes to metabolism of thiosulfate and sulphite was investigated by parallel incubation of wild type and mutated strain in TSI-agar (Oxoid CM0277, Thermo Scientific) and Iron-sulfite agar (Oxoid CM0079, Thermo Scientific) for 24 hours at 37°C.

### Induction of Membrane Stress by SDS

Growth was determined in presence of 0.01% (w/v) and 0.1% (w/v) SDS. Bacteria were grown to stationary phase in LB medium and adjusted to the same number. Bacteria were spotted on LB agar plates as previously described [46]. Prior to spotting the plates were adjusted to the test condition by addition of SDS, and growth was evaluated after 16–18 h of incubation at 37°C. As a control, growth on LB agar plates without SDS was followed in parallel. For control of plate assay, a broth assay was also performed with one of the concentrations. 20 ml of M9 media in 100 ml test tubes with 0.01% SDS was inoculated with colony material of each strain from an LB plate (OD_600_ value of 0.05), and growth of the bacteria at 37°C with shaking was followed.

### Resistance Towards Cyanide

The ability to detoxify cyanide was determined by growth in basis medium (in 1 liter containing 10 g peptone; 5 g NaCl; 0.225 g KH_2_PO_4_; 5.64 g Na_2_HPO_4_; pH = 7,4) supplemented with potassium cyanide (KCN) at the following concentrations: 0,3 mg/l; 0,6 mg/l; 3 mg/l; 15 mg/l; 75 mg/l. Media was inoculated with a colony of bacteria grown on LB agar, and incubated at 37°C. Growth was evaluated after two days of incubation. As a control, growth of each strain in non-supplemented basis medium was investigated in parallel.

### Resistance Towards H_2_O_2_

Strains were inoculated in LB media and incubated overnight at 37°C. The next day a dilution with PBS was made to OD_600_ = 0.2. The exact CFU (T0) was determined by plating in duplicate on LA agar using the glass bead method. H_2_O_2_ was added to a concentration of 10 mM, and CFU was determined at times T1, T2, T3 and T6 hours as mentioned for T0.

### Resistance to NO Stress

Resistance to NO stress was tested in growth experiments in the presence of S-Nitrosoglutathione (GSNO; Sigma-Aldrich) and in survival experiments after exposure to peroxynitrite (Caymen Chemicals). To determine the exact concentration of peroxinitrite, absorbance at 302 nm (A) was measured and the concentration C (C = A/E) was calculated based on the extinction coefficient ε = 1670 M^−1^ cm^−1^.

For growth experiments in the presence of GSNO, stationary phase bacterial cultures were adjusted to an optical density (OD) at 600nm of 0.005 in fresh M9 medium supplemented with 0.1 mM, 0.25 mM, 0.5 mM and 1 mM GSNO. Growth was performed in 96-well plates and followed over a period of 20 h by OD_600nm_ measurements in a microplate spectrophotometer (PowerWave XS, Biotek) with intermediate shaking of the plate.

For survival studies, logarithmically grown cultures in M9 (OD_600nm_ = 0.04±0.01) were treated with 360 µM peroxynitrite and samples were taken after 0 and 15 min. To determine the number of bacteria in the samples, serial dilutions were plated on LB agar plates. Survival of bacteria was determined by calculating the number of bacteria as colony forming units (CFU) per ml (CFU ml^−1^) after 15 min in relation to the number of bacteria at the beginning of the experiment. In growth and survival experiments in the presence of NO stress, two non-treated controls were investigated in parallel: one in non-supplemented medium and one in medium diluted with the corresponding compound solvent (0.3 M NaOH for peroxynitrite and distilled H_2_0 for GSNO experiments = 1∶10 diluted M9).

### Epithelial Cell Infection

Invasion of cultured epithelial INT-407 cells was investigated using a Gentamicin protection approach as described previously [47] with few modifications. In brief INT-407 cells were grown in MEM+Glutamax™-I, Earles, 25 mM HEPES (Gibco) supplemented with 10% (v/v) heat-inactivated fetal bovine serum (FBS; Invitrogen) at 37°C in an atmosphere containing 5% CO_2_. 18–20 h prior to infection, cells were seeded in 24-well plates at 1×10^5^ cells per well. Bacteria grown to stationary phase were re-grown to logarithmic phase in M9 (OD_600nm_ = 0.4±0.01) and were added to INT-407 cells at a multiplicity of infection (MOI) of 100. After 15 min of infection at 37°C and 5% CO_2_, cells were treated with 100 µg ml^−1^ gentamicin for 1 h to kill extracellular bacteria and dissolved in 0.1% (v/v) Triton X-100. Serial dilutions of the lysates were spread on LB agar plates to determine the number of invaded bacteria as CFU ml^−1^. To adjust for day to day variation, values were adjusted against CFU ml^−1^ of the wild type. In parallel bacterial enumerations of the inoculum was determined to ensure equal starting numbers.

### Infection of Macrophages

Cell culture experiments with J774.1A macrophages were performed essentially as previously described [48]. In brief J774.1A cells were grown in RPMI1640+Glutamax™-I, 25 mM HEPES (Gibco), supplemented with 10% (v/v) heat-inactivated FBS at 37°C and 5% CO_2_. 20–24 h prior to infection, cells were seeded in 24-well plates at 4×10^5^ cells per well. Bacteria grown to stationary phase in M9 were harvested at 8000 rpm for 5 min, re-suspended in 0.9% (w/v) NaCl, complement-opsonized and added to the cells at a MOI of 10∶1. A separate experiment was set up to demonstrate that the assay was capable of showing differences in survival capability between strains. This assay employed the wild type strain 14028 and an isogenic strain with insertion mutation of the SPI-2 gene *ssaV*, which causes attenuation for intra cellular propagation [49]. After 25 min of infection at 37°C, 5% CO_2_ (designated time zero p.i.), cells were further incubated in the presence of 100 µg ml^−1^ gentamicin for 1 h. The cells were either dissolved in 0.1% (v/v) Triton-X 100 (intracellular survival 1 h p.i.), or further incubated in the presence of 25 µg ml^−1^ gentamicin for a total incubation time of 4 h and 24 h (intracellular survival/replication 4 h and 24 h p.i.), and then dissolved as described above. In the control experiment, survival/replication was only followed for 20 hours. Serial dilutions were plated on LB agar plates to determine bacterial numbers of the inoculum (to ensure equal starting numbers) and the lysates as CFU ml^−1^. Values determined at T4 and T24 were expressed relatively to CFU for the WT determined at T1.

### Cytotoxicity Towards Macrophages

Cytotoxicity of bacteria towards macrophages was determined by measuring release of the lactate dehydrogenase (LDH). Infection studies with J774.1A macrophages and bacteria grown to stationary phase in M9 were performed as described above. 24 h p.i., supernatants were collected and LDH release was determined using the CytoTox 96®Non-Radioactive Cytotoxicity Assay kit (Promega) following the supplier instructions. Cytotoxicity was calculated as the percentage of LDH release in infected cells in relation to LDH release in non-infected, enzymatically lysed cells (maximum release).

### Mouse Infection

The ability to infect female six-week old C57/BL6 mice was assed as described previously [42]. Essentially mice were inoculated i.p. with a 50∶50 mixture of wild type and mutant bacteria at a challenge dose of 5×10^3^ CFU of each strain. The number of bacteria recovered from the spleen after 4 to 6 days of infection (variation of up to 30 hours was introduced between the time where mice were sacrificed because some animals had to be sacrificed earlier than day 6 due to severe illness to comply with the ethical clearance) was between 1×10^7^ CFU ml^−1^ and 5×10^8^ CFU ml^−1^. A few samples showed counts below 10^6^ CFU ml^−1^ and these were not included in the analysis. The competitive method used has been reported to be more sensitive for testing of virulence than individual challenge of strains, since the mouse-to-mouse variation is eliminated [50]. This lowers the need for many mice in each group. Competition indices were calculated based on input (CFU ml^−1^ of inoculum) and output (CFU ml^−1^ of spleen sample) numbers of wild type versus mutant bacteria as described previously [42]. Mice infection studies were performed with permission to John Elmerdahl Olsen from the Danish Animal Experiments Inspectorate, license number 2009/561-1675.

### Statistical Analyses

Statistical significance of the differences between wild type and mutant strains was determined using GraphPad Prism®, version 5.0 (GraphPad software) with one-sample t-test analysis. Grubb’s outlier test was performed to exclude outliers with a significance of 0.05.

## Results and Discussion

PspE is a member of the Psp system, which helps *S*. Typhimurium to cope with membrane stress (reviewed in [27]–[28], [51]). Recently, the first protein encoded in the *pspABCDE* operon, PspA, was demonstrated to be required for virulence in the mouse model of systemic infection [52]. However, *pspE* expression is predicted to occur independently from a *pspE*-specific promoter [29] and it is highly expressed during cell infection [31]–[32]. Therefore, we wanted to investigate the role of PspE in virulence in *S*. Typhimurium independent from the contribution of the remaining part of the Psp system. Furthermore, in order to investigate whether it has functional overlap with another TST, GlpE, as it does in *E. coli* [24], we also investigated the role this single domain TST in parallel. We generated *glpE* and *pspE* single and double knockout mutant strains in *S*. Typhimurium 4/74 as well as *in trans* complemented strains (Table 1) and characterized all strains. qPCR was used to measure expression of the two genes in wild type, mutated and complemented strains. Wild type and complemented strains expressed the genes in the expected way, while no expression was observed in mutated strains (data not shown).

### GlpE and PspE have a Role in Systemic Disease in the Mouse Model

In order to investigate whether GlpE and PspE have a role in virulence, we tested *glpE* and *pspE* mutant strains in competition with the wild type strain in a mouse model of systemic infection. Deletion of *pspE* increased virulence in C57/BL6 mice slightly (CI: 1.14; p<0.01). Introduction of the gene to the mutant *in trans* on the plasmid pINS02 removed significant differences between the two strains, suggesting that the increase in virulence was indeed caused by the *pspE* mutation. In contrast, deletion of *glpE* did not cause a significant change in virulence (Table 3). Interestingly, the Δ*glpE*/Δ*pspE* double mutant strain showed significantly decreased virulence (CI: 0.69±0.10; p<0.01), and introduction of the plasmid pINS04, encoding cloned copies of the two genes restored virulence to wild type level. Thus, the combined lack of GlpE and PspE decreased the ability of *S.* Typhimurium to carry out systemic disease in the mouse model, suggesting a role for TST in the infection process and complementary function of the two TSTs during the infection.

**Table 3:** Competitive indices of *S*. Typhimurium 4/74 wild type bacteria relative to mutant bacteria in the mouse spleen.

4/74 wild type versus	C.I. ± STD
ΔglpE (6)	0.80±0.25
ΔpspE (4)	1.14±0.03**
ΔpspE+pINS02 (6)	0.72±0.47
ΔglpE/ΔpspE (4)	0.69±0.10**
ΔglpE/ΔpspE+pINS04 (4)	0.97±0.25

C57/BL6 mice were infected i.p. with equal numbers of mutant and wild type bacteria (each 5×10^3^ CFU). After 4 to 6 days, mice were sacrificed and the spleen was removed. Serial dilutions were spotted on LB agar plates and number of wild type and mutant bacteria in a total of 100 colonies was further determined by selection of the resistance marker. Competitive indices (C.I.) were calculated as previously described [42]. The results are shown as mean values ± STD based on the number of mice tested as indicated in brackets. Significant differences from 1.0 (**p<0.01) were determined by one-sample t-test analysis.

The method used for virulence testing, i.e. competitive testing of wild type and mutant in the same animal was developed by Beuzon *et al.* [50] as a way to increase sensitivity in virulence testing. Since both wild type and mutant strain are tested in the same mice, the mouse-to-mouse variation in susceptibility to *Salmonella* infection is eliminated. As a consequence, fewer mice are needed to obtain the same statistical power as testing of each strain individually, which is in line with the international agreement to reduce the number of experimental animals used for research.

### Growth Phenotypes of GlpE and PspE Mutants of *S*. Typhimurium

**Figure 1:**
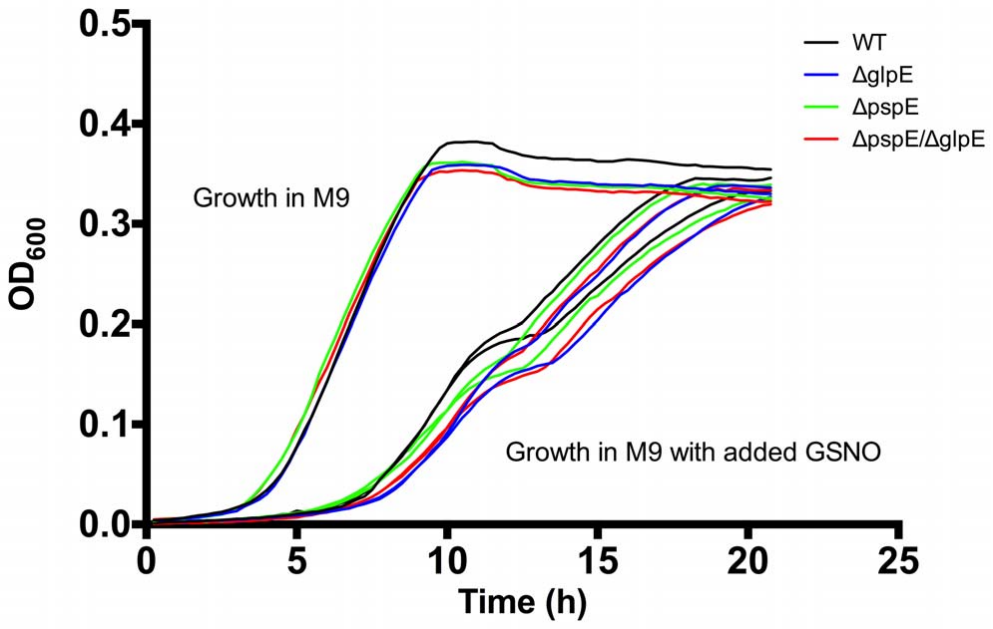
Growth of wild type and *glpE* and *pspE* mutant strains in M9 medium with and without GSNO. Bacteria grown to stationary phase in M9 were re-grown in M9 medium without supplement and in media with varying concentrations of GSNO. The figure shows results for growth in M9, M9+0.5 mM GSNO and M9+1 mM GSNO. Growth of the wild type (black), Δ*glpE* (blue), Δ*pspE* (green) and Δ*glpE*/Δ*pspE* (red) strains was monitored for 20 h at 37°C with intermediate shaking of the plate. The data show representative results of two independent replicates. Growth experiments showed similar growth of Δ*glpE*, Δ*pspE* and Δ*glpE*/Δ*pspE* strains compared to their isogenic wild type strain.

To rule out that the virulence phenotype was caused by a simple growth defect, we investigated whether GlpE and PspE mutants grew similar to the wild type strain in rich (LB) (data not shown) and minimal (M9+glucose) medium (Figure 1). Single as well as the double mutant showed similar growth curves as the wild type strain. TSTs are believed to assist in sulfur metabolism [13]. This also tested whether *glpE* and *pspE* are needed for the synthesis of sulfur containing amino acids, since the M9 medium contained MgSO_4_ as the only sulfur source, i.e. mutants were not affected in growth with MgSO_4_ as the only sulfur source. We also tested the ability of the strains to reduce thiosulfate and sulfite and also here we did not observe difference between the wild type and mutated strains, i.e all strains showed the typical ability of *Salmonella* to reduce these substances (data not shown), indicating that *glpE* and *pspE* are dispensable for the synthesis of essential sulfur containing amino acids in *S*. Typhimurium and for metabolism of thiosulfate and sulfite.

### GlpE and PspE Mutants are Dispensable During SDS Induced Membrane Stress

**Figure 2:**
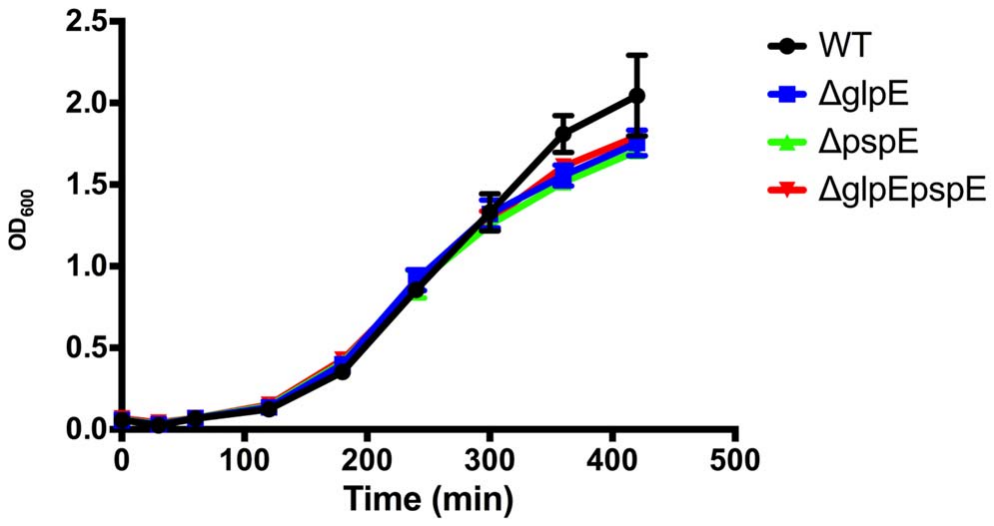
Growth of wild type 4/74 and Δ*pspE*, Δ*glpE* and Δ*pspE/glpE* strains in the presence 0.01% SDS. Strains were grown in 100 ml flasks containing 20 ml M9 supplemented with 0.01% SDS. The growth of wild type and mutated strains was similar.

Since the Psp system is believed to aid in counteracting membrane stress [26]–[27], we investigated whether the mutants would show increased sensitivity to SDS, which is the prototype stress factor for detergent shock proteins [53]. The 4/74 Δ*pspE*, Δ*glpE* and Δ*pspE/glpE* strains grew equally well as the wild type strain in the presence of 0.01% and 0.1% SDS (data not shown). Growth control on plates without SDS was included, and comparison to this showed that the conditions tested affected the growth of the wild type strain, showing that it indeed experienced a stress. The plate assay used was less sensitive than comparative growth experiments, and to further substantiate our conclusions, growth in the presence of 0.01% SDS was also performed in M9 media in 100 ml flasks. As seen in Figure 2, no difference was observed between wild type and mutated strains.

### GlpE and PspE are Dispensable for Resistance Towards NO stress *in vitro*

**Figure 3:**
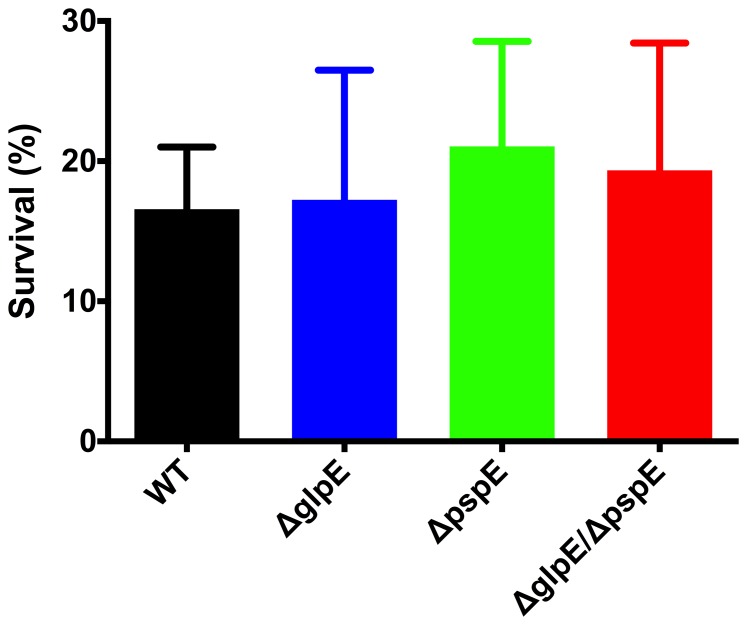
Survival of wild type 4/74 and Δ*pspE*, Δ*glpE* and Δ*pspE/glpE* strains in the presence of peroxynitrite treatment. Bacteria grown to logarithmic phase (OD_600nm_ = 0.04±0.01) were treated with 360 µM peroxynitrite for 15 min. Survival of bacteria was determined by calculating the number of bacteria after 15 min in relation to the number of bacteria at the beginning of the experiment. Results show mean values of at least three independent experiments ± SEM.

Nitric oxide compounds produced by the host are believed to interfere with important iron-sulfur complexes in bacteria [6]. *S.* Typhimurium has been reported to contain four iron-storage proteins, of which ferritin B encoded by *ftnB* and regulated by the Fur-system [54] has been identified as important for repair of iron-sulfur clusters [55]. Sulfur transferases are believed to be involved in the synthesis and repair of iron-sulfur clusters [13] as the bovine liver rhodanese is able to re-constitute iron-sulfur clusters of various enzymes *in vitro* [18]–[21]. We hypothesized that GlpE and PspE might be important for growth and survival in the presence of NO and RNS. To test this, we performed growth experiments of 4/74 wild type and mutant strains in the presence of GSNO and survival experiments after exposure to peroxinitrite. GSNO is a NO donor that primarily reacts with thiols, causing nitrosylation of proteins [9]. Peroxynitrite is a RNS that usually is formed in the cell by the reaction of NO with superoxide anion and which reacts with metal centers. Addition of GSNO at concentrations ranging from 0.1 to 1 mM lowered growth of the wild type strain in M9 compared to growth in the control medium without GSNO addition (Figure 1), showing that the growth reducing effect was indeed a result of GSNO addition. Growth inhibition was, however, similar between the wild type and the *glpE* and *pspE* mutant strains. From the experiments performed we cannot rule out that mutant specific responses would have been observed at higher concentrations. Furthermore, survival of the Δ*glpE*, Δ*pspE* and Δ*glpE*/Δ*pspE* strains in the presence of 360 µM peroxinitrite for 15 min likewise was similar to survival of the wild type strain (Fig. 3). Altogether, GlpE and PspE were concluded not to be required for resistance of *S.* Typhimurium to NO stress *in vitro* within the concentration range tested.

### *glpE* but not *pspE* Contributes Significantly to Resistance Towards H_2_O_2_

**Figure 4:**
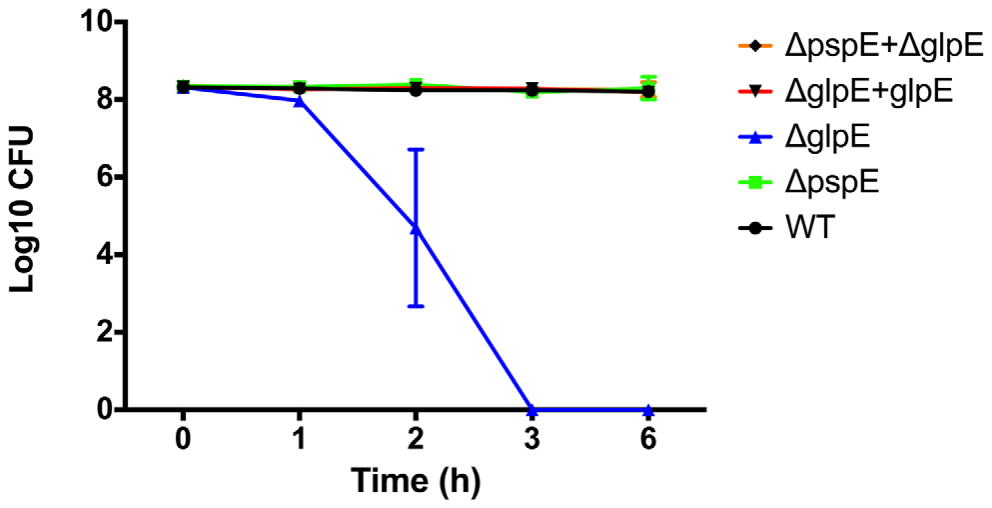
Growth of WT (4/74) and *pspE* and *glpE* mutated and complemented strains in M9 medium with 10 mM H_2_O_2_. Wild type (black), Δ*glpE* (blue), Δ*pspE* (green), Δ*glpE*/Δ*pspE* (red) and Δ*glpE*+*glpE* strains were inoculated to an OD_600_ value of 0.05, H_2_O_2_ was added and growth was followed for 16 hours. Results shown are representative of two biological repeats.

Like nitric oxide, H_2_O_2_ also affects the cell through damage of iron- clusters [56], and we found it indicated to investigate the role of GlpE and PspE in the protection against this oxidative stress molecule. We grew our mutants in the presence of 5 mM and 10 mM H_2_O_2_ in LB and M9 media and observed that the wild type strain was slightly affected in growth and that the Δ*glpE,* but not the Δ*pspE* mutant, was severely affected in growth under this condition in both media (growth in 10 mM H_2_O_2_ shown in Figure 4). The phenotype was fully complemented by addition of the wild type gene *in trans*. The role of GlpE in oxidative stress adaptation has not previously been investigated, and this observation is the first clear phenotype associated with GlpE in *S.* Typhimurium. Unexpectedly, the double Δ*glpEpspE* mutant was not affected (Figure 4). The reason for this remains elusive, but the observation was very reproducible and may indicate that the lack of GlpE is only critical for resistance to H_2_O_2_ in the presence of a fully functional PspE.

### *pspE* and *glpE* are not Important for *S.* Typhimurium Resistance Towards Cyanide

Another possible physiological role of sulfur transferases is the detoxification of cyanide as shown for the RhdA rhodanese in *Pseudomonas aeruginosa* [57]. In a standard biochemical classification *S. enterica* serovars are classified as non-detoxifying bacteria of cyanide at a concentration of 75 mg/l KCN [58]. However, we speculated that *glpE* and *pspE* might contribute to cyanide resistance at concentrations below this threshold. To test this, *S*. Typhimurium wild type, *glpE* and *pspE* single and double mutant strains were grown in the presence of KCN at concentrations ranging from 0.3 mg/l to 75 mg/l. The wild type and the Δ*glpE*, Δ*pspE* and Δ*glpE*/Δ*pspE* strains showed similar sensitivity towards KCN with the expected growth inhibition at 75 mg/l, poor growth at 15 mg/l and normal growth at 3 mg/l, 0.6 mg/l and 0.3 mg/l (data not shown), indicating that GlpE and PspE proteins are not involved in cyanide tolerance in *S.* Typhimurium.

### Intracellular Survival and Cytotoxicity Towards Macrophages is Independent of GlpE and PspE

**Figure 5:**
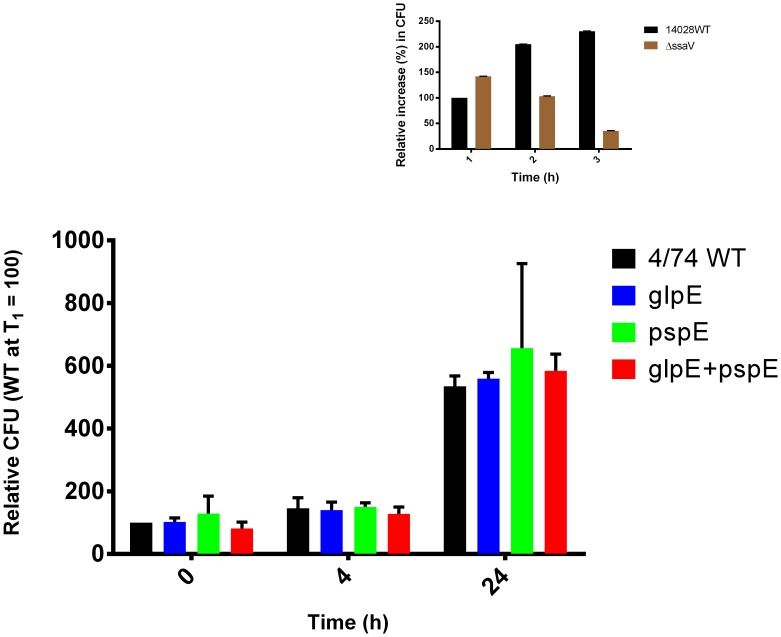
Infection of J774 macrophages with wild type 4/74 and Δ*pspE*, Δ*glpE* and Δ*pspE/glpE* strains. J774A.1 macrophages were infected with complemented-opsonized bacteria in a MOI of 10 and incubated for 25 min. Extracellular bacteria were killed by treatment with 100 µg ml^−1^ gentamicin. Bacteria were released from the macrophages 1 h p.i., 4 h p.i. and 24 h p.i. and the number of intracellular bacteria was determined by CFU ml^−1^ calculations and was expressed relatively to CFU at T1. The results show mean values ± SEM of at least three independent replicates. The small inserts shows results of an internal control experiment conducted with a SPI-2 (*ΔssaV*) deficient strain showing the expected reduced intracellular survival/replication of the mutated strain.

Virulence of *S*. Typhimurium in the mouse model of systemic disease was decreased in the absence of *glpE* and *pspE* (Table 3). In order to determine what might have caused this reduction, we tested the ability of *glpE* and *pspE* deficient and complemented strains to infect and survive inside J774 macrophages. This was considered relevant as these features play a role in the development of systemic disease of *S*. Typhimurium [1]–[2] and as both genes are expressed during infection of cultured macrophages [31]. Survival/replication inside J774 macrophages 1h p.i., 4h p.i. and 24 h p.i. was found to be similar to the wild type strain (Figure 5). A mutant in the SPI-2 gene *ssaV* which is attenuated for macrophage survival [49] was included as control, and showed the expected phenotype, as it was taken up to the same extend as the wild type strain, but showed reduced intracellular propagation. Theoretically, since cytotoxic effects results in exposure to gentamicin in such cell culture experiments, a strain with increased multiplication ability could be masked by an increase in cytotoxicity. However, the mutated strains did not differ significantly from the wild type in cytotoxicity 24 hours post infection (data not shown). Thus, *S*. Typhimurium intracellular survival and replication in macrophages was independent of the presence of the two putative TSTs GlpE and PspE. A possible explanation for the discrepancies in the *in vivo* and *in vitro* virulence data could be the limitations in use of cell culture experiments to study the complex interaction of *S*. Typhimurium with host cells and tissues [59]–[60].

### GlpE and PspE are also Dispensable for Invasion of Epithelial Cells

**Figure 6:**
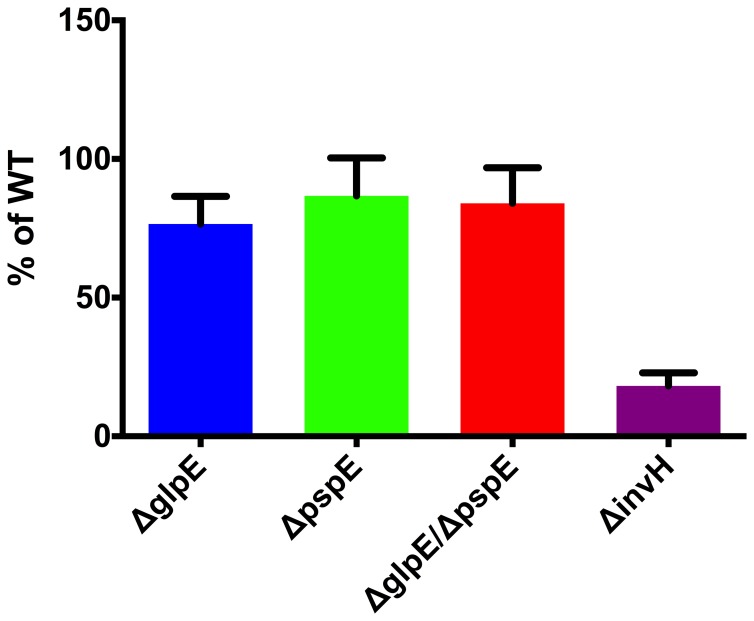
Infection of epithelial INT-407 cells wild type 4/74 and Δ*pspE*, Δ*glpE* and Δ*pspE/glpE* strains. Monolayers of INT-407 cells were infected with bacteria grown to logarithmic phase in a MOI of 100 and incubated for 15 min. Extracellular bacteria were killed by treatment with 100 µg ml^−1^ gentamicin. Number of intracellular bacteria was determined by CFU ml^−1^ calculations and values were adjusted against values for the wild type. The results represent mean values of at least three independent experiments ± SEM. Significant difference (**p<0.01) was determined by one-sample t-test analysis.

Infection of the intestinal epithelial layer is the first critical step in *Salmonella* virulence. This aspect of infection was bypassed in our mice experiments, since we used intra peritoneal challenge. The ability of *S*. Typhimurium to infect epithelial cells largely depends on expression of the SPI-1 encoded T3SS (T3SS1) and release of effector molecules through this system [1]. During infection of epithelial cells, *glpE* is constitutive and *pspE* is highly expressed [32]. We therefore tested the role of GlpE and PspE in invasion in an epithelial cell infection model. Moreover, we tested the ability of *glpE* and *pspE* mutant strains to express genes encoding regulatory, structural and effector molecules of the T3SS1. Single and double deletion of *glpE* and *pspE* genes did not change the ability of *S*. Typhimurium to invade epithelial INT-407 cells compared to the wild type, whereas the control strain Δ*invH*, an *S*. Typhimurium strain with a deficiency in T3SS1 [37], was decreased in this phenotype (p<0.01) (Figure 6). In line with these findings, single or double deletion of *glpE* and *pspE* genes in *S*. Typhimurium 4/74 did not change expression of *hilA*, *invG*, *prgH* and *sopB* genes compared to the wild type as determined from qPCR experiments (data not shown). The growth conditions we used to demonstrate this were not optimal for induction of SPI-1, but the genes have previously been demonstrated to be expressed under this condition [42]. Overall, *glpE* and *pspE* are dispensable for *in vitro* invasion of *S*. Typhimurium and expression of genes that are associated with the T3SS1.

## Conclusion

This worked revealed that parallel deletion of *glpE* and *pspE* genes decreased virulence of *S*. Typhimurium in the mouse model of typhoid fever, suggesting a role of TST activity in systemic infection. Deletion of *glpE* but not *pspE* significantly affected H_2_O_2_ resistance, but since the double mutant was not affected in this assay we found it unlikely that reduced oxidative stress was the reason for the virulence phenotype. Thus the mechanism by which GlpE and PspE contribute to virulence in *S*. Typhimurium remains to be characterized in future research.
